# Association of cytokine and matrix metalloproteinase profiles with disease activity and function in ankylosing spondylitis

**DOI:** 10.1186/ar3857

**Published:** 2012-05-28

**Authors:** Derek L Mattey, Jonathan C Packham, Nicola B Nixon, Lucy Coates, Paul Creamer, Sarah Hailwood, Gordon J Taylor, Ashok K Bhalla

**Affiliations:** 1 Haywood Rheumatology Centre, University Hospital of North Staffordshire, Stoke-on-Trent ST6 7AG, UK; 2 Institute of Science and Technology in Medicine, Keele University, Keele ST5 5BG, UK; 3 Royal National Hospital for Rheumatic Diseases, Bath BA1 1RL, UK; 4 Department of Rheumatology, Southmead Hospital, Bristol BS10 5NB, UK; 5 Department of Rheumatology, University Hospital of North Durham, Durham DH1 5TW, UK

## Abstract

**Introduction:**

The pathology of ankylosing spondylitis (AS) suggests that certain cytokines and matrix metalloproteinases (MMPs) might provide useful markers of disease activity. Serum levels of some cytokines and MMPs have been found to be elevated in active disease, but there is a general lack of information about biomarker profiles in AS and how these are related to disease activity and function. The purpose of this study was to investigate whether clinical measures of disease activity and function in AS are associated with particular profiles of circulating cytokines and MMPs.

**Methods:**

Measurement of 30 cytokines, five MMPs and four tissue inhibitors of metalloproteinases was carried out using Luminex^® ^technology on a well-characterised population of AS patients (*n *= 157). The relationship between biomarker levels and measures of disease activity (Bath ankylosing spondylitis disease activity index (BASDAI)), function (Bath ankylosing spondylitis functional index) and global health (Bath ankylosing spondylitis global health) was investigated. Principal component analysis was used to reduce the large number of biomarkers to a smaller set of independent components, which were investigated for their association with clinical measures. Further analyses were carried out using hierarchical clustering, multiple regression or multivariate logistic regression.

**Results:**

Principal component analysis identified eight clusters consisting of various combinations of cytokines and MMPs. The strongest association with the BASDAI was found with a component consisting of MMP-8, MMP-9, hepatocyte growth factor and CXCL8, and was independent of C-reactive protein levels. This component was also associated with current smoking. Hierarchical clustering revealed two distinct patient clusters that could be separated on the basis of MMP levels. The high MMP cluster was associated with increased C-reactive protein, the BASDAI and the Bath ankylosing spondylitis functional index.

**Conclusions:**

A profile consisting of high levels of MMP-8, MMP-9, hepatocyte growth factor and CXCL8 is associated with increased disease activity in AS. High MMP levels are also associated with smoking and worse function in AS.

## Introduction

Ankylosing spondylitis (AS) is a chronic inflammatory disease of the spine, three times more common in men than in women. It is characterised by sacroiliitis, accompanied by inflammation of the entheses and the spine [1]. Involvement of peripheral joints occurs in about 30% of patients. Extraskeletal manifestations include acute anterior uveitis, inflammatory bowel symptoms and renal, pulmonary and cardiac involvement. Although genetic factors are important in disease development, there is no clear way of predicting which patients will suffer severe disease [2].

Standard measures of acute phase response (erythrocyte sedimentation rate/C-reactive protein (CRP)) are not generally felt to be reliable indicators of activity of spinal disease [3]: these measures correlate weakly with disease activity in AS, do not fully reflect the disease process, and their value in clinical trials is limited. Neither measure appears to be clearly superior in terms of validity [3].

Although the serum levels of some cytokines and matrix metalloproteinases (MMPs) have been found to be elevated in active disease, there is a general lack of information about biomarker profiles in AS and how these are related to disease activity. Individual cytokines/cytokine receptors that have been associated with disease activity in AS include IL-6, transforming growth factor beta-1, vascular endothelial growth factor (VEGF), macrophage colony-stimulating factor and soluble IL-2 receptor [4-9]. Reductions in the circulating levels of transforming growth factor beta-1, IL-6, VEGF and macrophage colony-stimulating factor have been seen in patients treated with TNF inhibitors [10-14], although not all studies have shown changes after anti-TNF treatment [9,15].

Recent studies have suggested that serum MMP-3 is a useful marker of disease activity in AS, particularly in patients with peripheral synovitis [9,16,17]. A number of studies have shown that MMP-3 levels are reduced in response to TNF antagonists, although its usefulness as a marker of response is debatable [9,13,16,18-22]. A recent study has suggested that MMP-3 levels were not useful for monitoring and predicting response to etanercept in terms of disease activity and functional assessments [22]. However, higher levels of MMP-3 have been shown to be predictive of greater radiographic progression in AS, especially in patients with pre-existing damage [23].

Clinical disease activity and particular disease features in AS will possibly be associated with discrete cytokine profiles, and combinations of cytokines and associated markers (for example, MMPs) may be more informative than individual markers. With the availability of technology for measuring many different biomarkers simultaneously from small serum samples, we have carried out an investigation of the relationship between a large panel of cytokines/MMPs/tissue inhibitors of metalloproteinases (TIMPs) and disease activity in a well-characterised population of patients with established AS.

## Materials and methods

Patients with AS (*n *= 180) were recruited from six secondary care rheumatology centres in the UK (Bath, North Bristol, Cannock, Durham, Romford and Stoke-on-Trent). Patients invited to take part in the study were over 21 years of age, had AS according to the modified New York Criteria of 1984 [24], and provided written informed consent according to the declaration of Helsinki. The Trent Research Ethics Committee and the six site-specific National Health Service trusts approved the multicentre study. The majority of patients had been on a stable dose of nonsteroidal anti-inflammatory drugs for at least the previous 3 months. Patients on anti-TNF therapy, systemic steroid use in the previous 3 months or bisphosphonates in the previous 12 months were excluded from the study. Other exclusions included current use of methotrexate, sulfasalazine, raloxifene, calcitonin, phenylbutazone or hormone replacement therapy.

The principal aim of this study was to evaluate the effect of bisphosphonate (alendronate) on global health (Bath ankylosing spondylitis global health (BAS-G)) in AS (Bisphosphonates in Ankylosing Spondylitis trial). Secondary aims were to determine whether there were changes in disease activity, function and bone status, and whether changes in biomarker levels were different between patients treated with or without alendronate. A preliminary report has been published, which indicated that alendronate had no significant effect on changes in CRP, cytokine and MMP levels [25]. A further aim of the study was to investigate the relationship between circulating biomarker levels and measures of disease activity and function in AS patients on standard nonsteroidal anti-inflammatory drug therapy. Data collected from the baseline visit are reported here.

Baseline assessment included questionnaires to assess disease activity (Bath ankylosing spondylitis disease activity index (BASDAI)), function (Bath ankylosing spondylitis functional index (BASFI)) and well-being (BAS-G). The BASDAI is based on six questions related to fatigue, spinal pain, peripheral arthritis, enthesitis and morning stiffness (both severity and duration). The BASFI is a set of 10 questions that consider activities related to functional anatomy and the patients' ability to cope with everyday life. The BAS-G consists of two questions that assess the effect of the disease on the patient's well-being. A 10-cm visual analogue scale is used to answer questions to the BASDAI, BASFI and BAS-G. All measures are scored between 0 and 10, with higher values indicating worse disease activity, function or well-being.

Blood samples were taken for measurement of cytokines, MMPs and TIMPs, as well as a full blood count, urea and electrolyte levels, serum calcium and CRP. Information was also collected on smoking status, smoking duration, average number of cigarettes smoked per day and age of smoking cessation. Pack years were calculated (1 pack year = 20 cigarettes/day for 1 year) to provide a quantitative measure of smoking history. The effects of smoking intensity were assessed by categorising participants according to pack-year history, as in previous studies in AS [26]: category 1, 0 pack years; category 2, 1 to 15 pack years; category 3, 16 to 30 pack years; and category 4, > 30 pack years.

### Measurement of cytokines, MMPs and TIMPs

Sera were separated from bloods collected in plain Becton Dickinson Vacutainer^® ^tubes (Becton Dickinson, Oxford, Oxfordshire, UK) at study entry. All sera were stored at -70°C until required. Measurement of the various cytokines, MMPs and TIMPs was performed using multiplex, bead-based (Luminex^®^) assays on a Bio-Plex™ 200 suspension array system (Bio-Rad Laboratories, Hemel Hempstead, Hertfordshire, UK). The levels of 30 cytokines (Human cytokine 30-plex panel; Life Technologies, Paisley, UK), five MMPs and four TIMPS (Fluorokine multi-analyte MMP and TIMP kits; R&D Systems Europe, Abingdon, UK) were measured in separate multiplex assays according to the manufacturers' instructions. The following biomarkers were measured: cytokines and cytokine receptors - IL-1β, IL-1 receptor antagonist, IL-2, IL-2R, IL-4, IL-5, IL-6, IL-7, IL-10, IL-12p70, IL-13, IL-15, IL-17, IFNα, IFNγ, TNFα, epidermal growth factor, basic fibroblast growth factor, VEGF, hepatocyte growth factor (HGF), granulocyte colony-stimulating factor and granulocyte-macrophage colony-stimulating factor; chemokines - CXCL8, CXCL10, Eotaxin, macrophage inflammatory protein-1α (CCL3), macrophage inflammatory protein-1β (CCL4), monokine induced by gamma interferon (CXCL9), monocyte chemotactic protein-1(CCL2) and regulated upon activation, normal T-cell expressed, and secreted (CCL5); MMPs - MMP-1, MMP-2, MMP-3, MMP-8 and MMP-9; and TIMPs - TIMP-1, TIMP-2, TIMP-3 and TIMP-4. High and low control samples were used in each assay. To test the reproducibility of MMP measurements we also measured the same samples using standard ELISA kits for MMP-3 and MMP-8 (R&D Systems).

### Statistical analysis

All data were tested for normality and the appropriate parametric or nonparametric tests were selected. Continuous data were expressed as mean ± standard deviation or median (interquartile range), as appropriate. Univariate correlations between biomarker levels and disease measures were carried out using Spearman's correlation. A multivariate variable selection procedure using the algorithm of McHenry [27] was initially used to select cytokine, MMP or TIMP variables that showed the strongest association with each clinical assessment. Multiple regression analysis or multivariate logistic regression analysis were used to investigate the association between clinical measures and biomarker levels while adjusting for other possible confounders. Where appropriate, data transformation to normality (log or square-root transformation) was carried out before analysis. When cytokine levels were below the level of detection, we carried out imputation of the lowest standard for that particular cytokine [28].

### Principal component analysis

Principal component analysis (PCA) is an exploratory technique that reduces the dimensionality of a large number of variables to a smaller set of uncorrelated independent components, and allows the identification of combinations of variables that best explain the differences between observations. PCA allows the identification of patterns within the variables and expresses them in a way that highlights the similarities and differences between them. Principal components (PCs) were extracted using varimax rotation, with the factor selection based on an eigenvalue cutoff of 1.0. The PCs identified were used in multivariate analysis to look for associations with clinical measures.

### Hierarchical cluster analysis

Biomarker levels were first converted to log_2 _and expressed relative to the normalised mean value. These measurements were used to generate heat maps using Genesis software (version 1.7.2; Alexander Sturn, Institute for Genomics and Bioinformatics, Graz University of Technology, Graz, Austria). The Genesis program uses a hierarchical clustering method that enables groups of variables with similar expression levels to be clustered together, as well as grouping together patient samples with similar expression patterns.

Statistical analyses were carried out using Number Cruncher Statistical Software package for Windows (Number Cruncher Statistical System 2000; NCSS Statistical Software, Kaysville, UT, USA). The significance level was set at *P *= 0.05.

## Results

### Characteristics of the ankylosing spondylitis patients

Table 1 displays the demographic and clinical characteristics of the 180 AS subjects from the six centres involved in the study. Serum samples were available on 157 patients at baseline. No difference was found in clinical disease measures between patients with and without serum samples available. Information on smoking status (never, past, current) was available for all patients investigated, although the pack-year history was only obtained for 68/96 (70.8%) of patients who had smoked.

**Table 1:** Demographic and clinical characteristics of the ankylosing spondylitis patients at baseline

Variable	All patients (*n *= 180)	Biomarker study (*n *= 157)
Age (years)	48.7 (37.0 to 56.5)	49.05 (38.3 to 57.7)
Duration (years)	18.0 (10.0 to 31.0)	18.0 (11.0 to 31.0)
Male	147/180 (81.7%)	130/157 (82.8%)
CRP (mg/dl)	7.9 (5.0 to 15.0)	7.0 (5.0 to 15.0)
BASDAI	4.04 (2.29 to 5.77)	4.00 (2.18 to 5.76)
BASFI	3.30 (1.80 to 5.38)	3.33 (1.78 to 5.43)
BASG	4.25 (2.12 to 6.20)	4.10 (2.15 to 6.20)
Ever smoked	106/180 (58.9%)	95/157 (63.8%)
Current smoker	47/180 (26.1%)	40/157 (25.5%)

Values are median (interquartile range) unless otherwise stated. BASDAI, Bath ankylosing spondylitis disease activity index; BASFI, Bath ankylosing spondylitis functional index; BAS-G, Bath ankylosing spondylitis global health; CRP, C-reactive protein.

### Correlation between serum biomarkers and clinical disease measures at baseline

Variables showing significant correlations (Spearman) are shown in Additional File 1. Baseline CRP levels correlated significantly with baseline levels of IL-6 (*r *= 0.25, *P *= 0.004), MMP-1 (*r *= 0.17, *P *= 0.04), MMP-2 (*r *= 0.17, *P *= 0.04), MMP-3 (*r *= 0.24, *P *= 0.002), MMP-8 (*r *= 0.23, *P *= 0.003) and MMP-9 (*r *= 0.23, *P *= 0.003). Baseline CRP was correlated with the BASFI (*r *= 0.19, *P *= 0.01) but not the BASDAI or BAS-G, and no correlation was found between IL-6 levels and the BASDAI, BASFI or BAS-G. Significant correlations with all three clinical assessments were found, however, for HGF and MMP-8 levels (*r *≥ 0.20, *P *≥ 0.02). For the BASDAI, significant correlations were also found with CXCL10 (*r *= 0.17, *P *= 0.04) and MMP-9 levels (*r *= 0.24, *P *= 0.002). MMP-9 levels were also correlated with the BASFI (*r *= 0.17, *P *= 0.03), while MMP-1 levels were correlated with both the BASFI and BAS-G (*r *= 0.20, *P *= 0.01 and *r *= 0.19, *P *= 0.01, respectively).

The reproducibility of MMP-3 and MMP-8 correlations with clinical measures was tested by also measuring these MMPs using standard ELISA kits (R&D Systems). The results were similar to those achieved using the Luminex^® ^system. MMP-3 levels were correlated with CRP levels but not with the BASDAI, BASFI or BAS-G. MMP-8 levels were significantly correlated with CRP and all three clinical assessments (Additional file 2).

### Multivariate analyses

A multivariate variable selection procedure was initially used to select cytokine, MMP or TIMP variables that showed the strongest association with each clinical assessment. To further determine which variables were independently associated with clinical measures we then carried out multiple regression analyses for each clinical assessment (dependent variable) in models that were adjusted for age, sex and disease duration, and included all significant variables found in the preliminary analyses.

### Associations with C-reactive protein

MMP-2, MMP-3 and MMP-8 levels were found to be independently associated with the CRP level (log transformed) at baseline (Table 2). MMP-3 and MMP-8 were positively associated while MMP-2 showed a negative association. No association of IL-6 or any other cytokine, MMP or TIMP was found in this model. Adjustment for age, sex and disease duration made little or no difference to the association of CRP levels with MMP levels. A similar model of association was found when MMP-3 and MMP-8 levels measured by ELISA replaced those obtained by Luminex^® ^analysis (data not shown).

**Table 2:** Multiple regression analysis showing variables associated with CRP levels in ankylosing spondylitis patients at baseline

	Response variable (CRP)^a^	
	
Independent variable	Regression coefficient (SE)	*P *value
MMP-3 (pg/ml)	1.108 × 10^-5 ^(2.335 × 10^-6^)	0.000005
MMP-8 (pg/ml)	4.189 × 10^-6 ^(1.610 × 10^-6^)	0.010
MMP-2 (pg/ml)	-2.277 × 10^-6 ^(9.477 × 10^-7^)	0.018

SE, standard error. ^a^C-reactive protein (CRP) levels were log transformed to normality before analysis.

### Associations with the BASDAI

Female sex and MMP-8 levels provided the best model for association with the BASDAI (square root transformed) at baseline (Table 3, Model 1), although a very similar model was found with female sex and MMP-9 levels as independent variables (Additional file 3). MMP-8 and MMP-9 levels were not independently associated since both lost significance in models containing both MMPs as independent variables. CRP was independently associated in a model adjusted for female sex only, but lost significance in models containing MMP-8 or MMP-9 levels. No other MMP, TIMP or cytokine were associated in these models. MMP-8 levels measured by ELISA were also significantly associated with the BASDAI in a model that also contained female sex (data not shown).

**Table 3:** Multiple regression models showing variables associated with the BASDAI, BASFI and BAS-G at baseline

	Response variable^a^	
	
Independent variable	Regression coefficient (SE)	*P *value
Model 1	BASDAI	
Female	0.302 (0.119)	0.012
MMP-8 (pg/ml)	6.667 × 10^-6 ^(2.533 × 10^-6^)	0.009
CRP (mg/l)	0.005 (0.003)	0.054
Model 2	BASFI	
Age	0.012 (0.004)	0.002
CRP (mg/dl)	0.011 (0.003)	0.001
Model 3	BAS-G	
Female	0.314 (0.131)	0.018
MMP-8 (pg/ml)	6.548 × 10^-6 ^(2.786 × 10^-6^)	0.020

CRP, C-reactive protein; MMP, matrix metalloproteinase; SE, standard error. ^a^For each regression model, square root transformation of the response variable - Bath ankylosing spondylitis disease activity index (BASDAI), Bath ankylosing spondylitis functional index (BASFI) or Bath ankylosing spondylitis global health (BAS-G) - was carried out to achieve a normal distribution of the data.

### Associations with the BASFI

The strongest association with the BASFI (square root transformed) was found in a model containing age and CRP levels as independent variables (Table 3, Model 2). There were no independent associations of any cytokine, MMP or TIMP with the BASFI in models that contained CRP.

### Associations with the BAS-G

As for the BASDAI, female sex and MMP-8 levels provided the strongest independent association with the BAS-G (Table 3, Model 3). In this case, MMP-8 levels remained independently associated in models that also contained MMP-9 as a variable (Additional file 3). A similar model was obtained when MMP-8 levels measured by ELISA replaced those obtained by Luminex^® ^analysis (data not shown). No association was found with CRP levels, although this approached significance (*P *= 0.07) in models that did not include MMP-8.

### Principal component analysis

We carried out PCA to investigate patterns of cytokines and MMPs in the data. This allowed the identification of factors containing particular biomarker profiles, which were examined for their association with clinical assessments in AS. Eight PCs were identified, explaining 78.3% of the total variance. These PCs are shown in Table 4 along with their respective factor loadings after varimax rotation.

**Table 4:** Principal component analysis of serum biomarkers in ankylosing spondylitis patients at baseline

PC1	PC2	PC3	PC4	PC5	PC6	PC7	PC8
IL-15 (0.972)	IFNγ (0.955)	MMP-8 (0.849)	GM-CSF (0.913)	CXCL10 (-0.676)	MMP-2 (-0.700)	bFGF (0.744)	EGF (0.735)
IL-2 (0.970)	IL-12 (0.936)	MMP-9 (0.796)	IL-10 (0.813)	IL-13 (0.626)	MMP-3 (-0.759)	IL-1Ra (0.493)	CXCL8 (0.436)
MIP-1β (0.930)	IL-5 (0.842)	CXCL8 (0.527)		VEGF (0.437)			MCP-1 (0.425)
IFNα (0.916)	Eotaxin (0.760)	HGF (0.453)					
IL-1β (0.877)	IL-2R (0.712)						
TNFα (0.841)	IL-17 (0.633)						
MIP-1α (0.812)	IL-4 (-0.565)						
IL-7 (0.771)	IL-13 (0.525)						
IL-6 (0.736)	MCP-1 (0.499)						
MIG (0.722)	VEGF (-0.477)						
IL-1Ra (0.680)	TNFα (0.408)						
IL-17 (0.674)							
IL-4 (0.673)							
IL-2R (0.609)							
GCSF (0.486)							
HGF (0.462)							
30.16%^a^	17.61%^a^	6.63%^a^	6.03%^a^	4.80%^a^	3.90%^a^	4.70%^a^	4.44%^a^

Biomarkers in each principal component are shown with factor loadings (brackets) after varimax rotation. bFGF, basic fibroblast growth factor; EGF, epidermal growth factor; G-CSF, granulocyte colony-stimulating factor; GM-CSF, granulocyte-macrophage colony-stimulating factor; HGF, hepatocyte growth factor; IL-1Ra, IL-1 receptor antagonist; MCP-1, monocyte chemotactic protein-1; MIG, monokine induced by gamma interferon; MIP-1, macrophage inflammatory protein-1; MMP, matrix metalloproteinase; PC, principal component; VEGF, vascular endothelial growth factor. ^a^Percentage variance.

The only PC showing an association with any clinical assessments was PC3, consisting of MMP-8, MMP-9, CXCL8 and HGF (Table 4). This component was associated with both CRP (log transformed) and the BASDAI (square root transformed). The latter association was independent of CRP, and remained significant after adjustment for age, sex and disease duration (Table 5). Interestingly, PC3 was also significantly higher in current smokers than in past smokers (0.455 vs. 0.089, *P *= 0.004) or in patients who had never smoked (0.455 vs. -0.350, *P *= 0.01). There was also a significant trend (*P *= 0.0005) of increasing PC3 level with increasing pack-year category (Table 6). Analysis of the individual markers of the PC3 profile demonstrated that MMP-8 and MMP-9 were both higher in current smokers than in past or nonsmokers (Table 7). MMP-8 levels also showed an increasing trend (*P *< 0.0001) with increasing pack-year category, although no trend was seen with MMP-9 levels (Additional file 4).

**Table 5:** Multiple regression analysis showing baseline association of PC3 (MMP-8, MMP-9, HGF, CXCL8) with the BASDAI

	Response variable (BASDAI)^a^	
	
Independent variable	Regression coefficient (SE)	*P *value
Female	0.282 (0.128)	0.029
PC3	0.080 (0.027)	0.004

HGF, hepatocyte growth factor; MMP, matrix metalloproteinase; PC, principal component; SE, standard error. ^a^Square root transformation of the Bath ankylosing spondylitis disease activity index (BASDAI) was carried out to achieve a normal distribution.

**Table 6:** Association between principal component 3 and pack-year category

Variable	0 pack years (*n *= 61	1 to 15 pack years (*n *= 37)	16 to 30 pack years (*n *= 17)	> 30 pack years (*n *= 14)	*P *(trend)^a^
PC3	-0.10 (1.80)	0.38 (1.17)	0.79 (1.21)	1.86 (2.51)	< 0.0005

Mean (standard deviation) values are shown. PC, principal component. ^a^Linear test for trend.

**Table 7:** Levels of individual biomarkers comprising principal component 3 stratified by smoking status

	Smoking status					
	
Variable (pg/ml)	Never (*n *= 61)	Past (*n *= 56)	Current (*n *= 40)	*P *value^a^	Ever (*n *= 96)	*P *value^b^
MMP-8	9,750 (4,767 to 14,668)	12,578 (6,906 to 26,066)	18,934 (10,792 to 28,988)	0.02	16,731 (8,001 to 27,241)	0.004
MMP-9	377,000 (258,488 to 607,190)	421,409 (248,758 to 627,056)	547,697 (344,692 to 802566)	0.01	466,416 (284,003 to 730,264)	0.04
CXCL8	27.0 (21.1 to 40.5)	30.0 (23.2 to 53.0)	34.0 (20.1 to 88.9)	0.4	31.9 (21.3 to 52.2)	0.2
HGF	421.7 (327.8 to 678.3)	430.3 (305.9 to 648.3)	408.4 (337.7 to 555.2)	1.0	427.1 (327.8 to 646.1)	0.9

HGF, hepatocyte growth factor; MMP, matrix metalloproteinase. ^a^Kruskal-Wallis test. ^b^Ever smoked versus never smoked (Mann-Whitney U test).

### Hierarchical clustering analysis

Hierarchical cluster analysis was carried out on cytokines, MMPs and TIMPS separately and in combination. Heat maps generated from these analyses were used to identify patient groups that clustered together according to the similarity or dissimilarity of their profiles.

**Figure 1:**
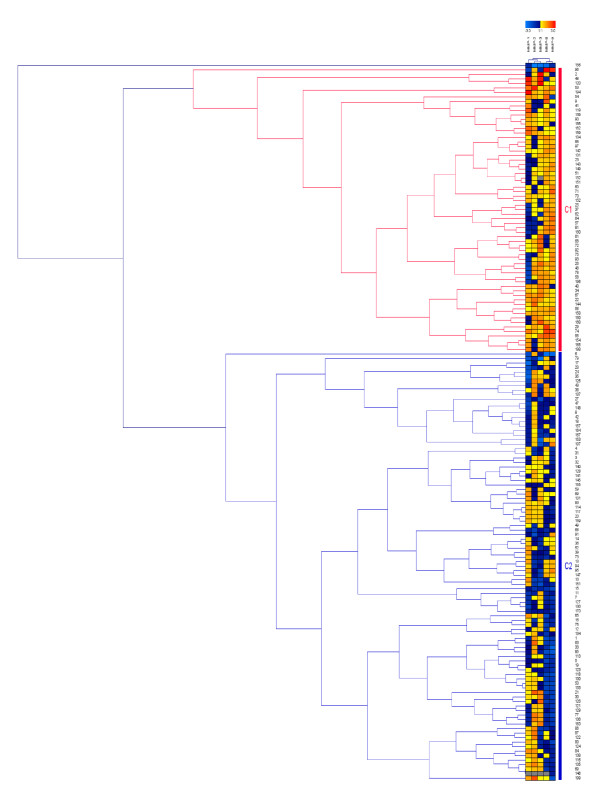
**Hierarchical cluster analysis of serum matrix metalloproteinase levels in patients with ankylosing spondylitis**. Results are displayed as a heat map and dendrogram in which the relative levels of matrix metalloproteinases (MMPs) are represented by shades of yellow/red (high) and blue (low). Each row represents the MMP profile for an individual patient, each of which is represented by a number on the vertical axis. Each column represents a different MMP (MMP-1, MMP-2, MMP-3, MMP-8 and MMP-9, left to right). C1, high MMP cluster; C2, low MMP cluster.

Discrimination between patient groups was best achieved by hierarchical cluster analysis of the five MMPs alone. This resulted in clustering of two major groups of patients based on relatively low or high MMP levels (Figure 1). The high MMP group was characterised by significantly higher levels of MMP-1 (20% higher), MMP-3 (43% higher), MMP-8 (222% higher) and MMP-9 (128% higher) (Additional file 5). There was no difference in MMP-2 levels between the two groups.

The high MMP group of patients had significantly higher CRP levels (median 12.0 vs. 6.0, *P *= 0.001), higher BASDAI scores (median, 4.67 vs. 3.27, *P *= 0.006) and higher BASFI scores (median, 3.83 vs. 2.94, *P *= 0.03), although the association with the BAS-G (median 5.0 vs. 3.6, *P *= 0.1) was not significant. The level of PC3 was also significantly higher in the high MMP group (1.125 vs. -0.511, *P *< 0.0001). In addition, patients in the high MMP group were more likely to be current smokers than those in the low MMP group (36.5% vs. 17.9%, odds ratio 2.60, 95% CI 1.26 to 5.38, *P *= 0.01). An increasing frequency of the high MMP group was also found with increasing pack-year category (*P *trend = 0.001) (Table 8).

**Table 8:** Relationship between pack-year category and frequency of AS patients in low and high MMP clusters

Pack years	Low MMP cluster	High MMP cluster
0	46 (73.0)	17 (27.0)
1 to 15	19 (52.8)	17 (47.2)
16 to 30	8 (47.1)	9 (52.9)
> 30	5 (35.7)	9 (64.3)^a^

Data presented as *n *(%). AS, ankylosing spondylitis; MMP, matrix metalloproteinase. *^a^P *(trend) = 0.001 (Cochrane-Armitage test for trend).

## Discussion

In this study we analysed the serum levels of a large panel of cytokines, chemokines, MMPs and TIMPs for their association with measures of disease activity and function in AS, using univariate and multivariate methods. We believe this is the largest study to date to examine such a large panel of biomarkers in AS. Our data indicate that serum levels of MMPs in particular show the strongest association with disease activity, as measured by the BASDAI. In contrast to some previous studies, we found that the strongest association was with MMP-8 (or MMP-9) levels, rather than MMP-3 levels. Importantly, we obtained similar findings using the Luminex^® ^system and ELISA methods. It is worth noting that previously reported relationships between MMP-3 levels and the BASDAI have often been based on univariate correlations rather than multivariate regression analysis, and correlations have not been consistently found in all studies [9,14,16,17,23]. This may reflect differences in the patient groups since it has also been suggested that MMP-3 levels are higher in patients with peripheral arthritis than those with axial disease only [14,16,17], although this has not always been observed [23]. The same may apply to IL-6, which has also been shown to be higher in patients with peripheral synovitis [5] but is not associated with the BASDAI in all studies [14]. One of the limitations of the present study was the absence of information on peripheral joint disease in the patients studied.

The correlations of MMP-3 and IL-6 with disease activity in some studies may be explained in part by the association of these molecules with CRP levels. Like previous studies, we found correlations of MMP-3 and IL-6 with CRP levels - although we also found that MMP-2 and MMP-8 levels were independently associated in a multivariate model containing MMP-3. IL-6 was not associated in this model. Interestingly the association with MMP-2 was negative, suggesting an anti-inflammatory role for this MMP, which is consistent with other studies in inflammatory arthritis [29,30].

The associations of MMP-8 and MMP-9 levels with the BASDAI were not independent of each other, and probably reflect involvement of the same pathway in the release of these particular MMPs. MMP-8 (neutrophil collagenase) is primarily produced by activated neutrophils, while MMP-9 is released mainly by neutrophils and macrophages.

In the PCA, MMP-8 and MMP-9 are also associated with CXCL8 and HGF (in the PC3 profile), both of which have also been associated with neutrophil activation [31,32]. The association of the BASDAI with this component may thus reflect a possible relationship between disease activity and neutrophil activation in AS. However, the biomarkers making up the PC3 profile are also associated with aspects of angiogenesis [33-35], although this may be linked also to an association with neutrophil activation. For example, neutrophil-derived MMPs can promote neoangiogenesis through release and activation of angiogenesis promoters (for example, VEGF-A) from the extracellular matrix [33]. Other studies in AS have shown a correlation between VEGF levels and disease activity [8,13,14], although we failed to find an association in this study. Further work is needed to determine with which particular aspects of the disease process the markers associated with PC3 are most closely associated. The association of PC3 with the BASDAI independent of CRP suggests that it is not merely a surrogate for systemic inflammation.

It is noteworthy that PC3 was also associated with current smoking, and that a significant trend was seen in relation to pack-year history. This appears to be largely due to associations with MMP-8 and MMP-9 levels, which have been shown in other studies to be increased in smokers [36,37]. Hierarchical clustering analysis also revealed an association between patients with high MMP levels and current smoking, as well as a quantitative relationship with pack-year history. All of these data add weight to the idea that the PC3 profile may reflect neutrophil activation, which is characteristically found in smokers [38,39]. A number of studies have indicated that smoking is associated with increased disease activity, worse functional outcome and poorer quality of life in patients with AS [26,40-43]. Smoking has also been associated with radiographic spinal progression in early axial spondyloarthritis independently of baseline radiographic damage and elevated acute phase reactants [44]. These associations may be explained in part by the increased levels of MMPs (particularly MMP-8 and MMP-9) in smokers. This association is suggested by the current study since the association of the BASDAI and BASFI with the pack-year category disappears when MMP-8 or MMP-9 levels are included in multivariate regression models along with pack-year history (data not shown). Smoking may thus exacerbate the production of neutrophil activation markers, but there also appears to be an association of these markers with disease activity independent of smoking.

The association of disease activity with markers of neutrophil and/or macrophage activation is interesting in light of studies suggesting that the innate immune pathway might be more important in axial spondyloarthritis than the adaptive immune response [45-48]. Infiltration of the synovium in axial spondyloarthritis by macrophage subsets and neutrophils has been shown to reflect global disease activity [47], while more recently the role of IL-17 in axial spondyloarthritis has been linked to increased numbers of IL-17^+ ^neutrophils and macrophage subsets in the subchondral bone marrow of affected facet joints [48]. Other cell types such as CD3^+ ^T cells, mast cells, B cells and natural killer cells provided little or no source of IL-17. The hypothesis that the innate immune pathway, mostly mediated through neutrophils, might be of greater relevance in AS inflammation than the T-helper type 17 cell-mediated adaptive immune response was therefore suggested. Such a suggestion is in line with earlier studies that indicated significant alterations in neutrophil function in patients with AS [49-54]. These include alterations in chemotaxis, phagocytosis and superoxide radical anion generation. Priming of neutrophils has been suggested to be a probable causative factor in the onset of AS, and increased production of reactive oxygen species and MMPs from these cells may lead to tissue damage in AS [53]. Increased levels of advanced oxidation protein products, a novel oxidative stress marker of protein, have been demonstrated in AS patients, and provide evidence of oxidative stress mediated by neutrophil myeloperoxidase-hypochlorous acid in these patients [54]. The cause of increased neutrophil activation in AS is unclear - although smoking is likely to have an exacerbating effect, and cessation of smoking may therefore provide one area for limited control of disease.

## Conclusion

We have shown, using several methods of analysis, that higher disease activity and worse function in patients with established AS are associated with increased serum levels of MMPs (particularly MMP-8 and MMP-9) and cytokines/chemokines (HGF, CXCL8) associated with neutrophil activation and/or angiogenesis. Serum MMP-8 and/or MMP-9 levels are more strongly associated with disease activity than MMP-3, and biomarker profiles containing high MMP-8/MMP-9 levels are associated with smoking in AS.

Further work is needed to investigate the role of these particular molecules in determining radiographic outcome in AS, and their potential use as markers of response to therapy.

## Abbreviations

AS: ankylosing spondylitis; BASDAI: Bath ankylosing spondylitis disease activity index; BASFI: Bath ankylosing spondylitis functional index; BAS-G: Bath ankylosing spondylitis global health; CRP: C-reactive protein; ELISA: enzyme-linked immunosorbent assay; HGF: hepatocyte growth factor; IFN: interferon; IL: interleukin; MMP: matrix metalloproteinase; PC: principal component; PCA: principal component analysis; TIMP: tissue inhibitor of metalloproteinase; TNF: tumour necrosis factor; VEGF: vascular endothelial growth factor.

## Competing interests

The authors declare that they have no competing interests.

## Authors' contributions

DLM and NBN carried out the biomarker measurements. DLM and GJT carried out the statistical analysis. JCP, LC, PC, SH and AKB participated in the design of the study, and recruitment of patients. DLM and JCP conceived the study, participated in its design and coordination, carried out analysis and interpretation of data, and drafted the final manuscript. All authors read and approved the final manuscript.

## Supplementary Material

Additional file 1 **Table S1 presenting correlations between clinical measures and biomarkers in ankylosing spondylitis patients at baseline**. Click here for file

Additional file 2 **Table S2 presenting correlations between clinical measures and MMP-3 and MMP-8 levels measured by ELISA in ankylosing spondylitis patients at baseline**. Click here for file

Additional file 3 **Table S3 presenting alternative multiple regression models showing variables associated with the BASDAI and BAS-G at baseline**. Click here for file

Additional file 4 **Table S4 presenting levels of individual biomarkers comprising principal component 3 stratified by pack-year category**. Click here for file

Additional file 5 **Table S5 presenting a comparison of MMP levels in patient clusters with low or high MMP levels as selected by hierarchical cluster analysis**. Click here for file
