# Supplementary material for: Association of cytokine and matrix metalloproteinase profiles with disease activity and function in ankylosing spondylitis
Source: Arthritis Res Ther. 2012 May 28;14(3):R127. doi: 10.1186/ar3857 (PMC3446508; doi:10.1186/ar3857)
Supplement: Additional file 1 — Table S1 presenting correlations between clinical measures and biomarkers in ankylosing spondylitis patients at baseline. [file ar3857-S1.PDF]

Table S1. Correlations between clinical measures and biomarkers in ankylosing spondylitis patients at baseline

	BASFI	BAS-G	CRP	MMP-1	MMP-2	MMP-3	MMP-8	MMP-9	IL-6	HGF	CXCL10
BASDAI	**0.762**	**0.781**	0.091	0.115	-0.051	-0.024	**0.295**	**0.244**	0.027	**0.202**	**0.172**
BASFI		**0.690**	**0.188**	**0.204**	0.095	0.014	**0.238**	**0.175**	0.085	**0.227**	0.142
BAS-G			0.066	**0.194**	0.049	-0.031	**0.250**	0.097	0.063	**0.230**	0.092
CRP				**0.167**	**-0.165**	**0.243**	**0.233**	**0.235**	**0.247**	0.086	0.029
MMP-1					0.040	0.089	0.086	0.054	0.011	0.139	0.068
MMP-2						**0.222**	-0.048	-0.161	0.068	0.079	0.192
MMP-3							0.131	0.136	0.133	0.163	-0.013
MMP-8								**0.665**	**0.187**	**0.302**	**0.225**
MMP-9									0.070	**0.377**	0.140
IL-6										**0.180**	**0.183**
HGF											**0.230**

Significant correlations (Spearman) are shown in bold.
