# Supplementary material for: Association of cytokine and matrix metalloproteinase profiles with disease activity and function in ankylosing spondylitis
Source: Arthritis Res Ther. 2012 May 28;14(3):R127. doi: 10.1186/ar3857 (PMC3446508; doi:10.1186/ar3857)
Supplement: Additional file 2 — Table S2 presenting correlations between clinical measures and MMP-3 and MMP-8 levels measured by ELISA in ankylosing spondylitis patients at baseline. [file ar3857-S2.PDF]

Table S2. Correlations between clinical measures and MMP-3 and MMP-8 levels measured by ELISA in ankylosing spondylitis patients at baseline

	BASFI	BAS-G	CRP	MMP-3	MMP-8
BASDAI	**0.762**	**0.781**	0.093	-0.001	**0.205**
BASFI		**0.690**	**0.188**	-0.040	**0.193**
BAS-G			0.066	-0.070	**0.196**
CRP				**0.275**	**0.213**
MMP-3					0.029

Significant correlations (Spearman) are shown in bold.
