# Supplementary material for: Association of cytokine and matrix metalloproteinase profiles with disease activity and function in ankylosing spondylitis
Source: Arthritis Res Ther. 2012 May 28;14(3):R127. doi: 10.1186/ar3857 (PMC3446508; doi:10.1186/ar3857)
Supplement: Additional file 3 — Table S3 presenting alternative multiple regression models showing variables associated with the BASDAI and BAS-G at baseline. [file ar3857-S3.PDF]

Table S3. Alternative multiple regression models showing variables associated with BASDAI and BAS-G at baseline

	Response variable[1]	
Independent variable	Regression coefficient (SE)	p value
Model 1	BASDAI	
Female	0.270 (0.117)	0.023
MMP-9 (pg/ml)	4.443×10^{-7} (1.565×10^{-7})	0.006
CRP (mg/l)	0.005 (0.003)	0.063
Model 2	BAS-G	
Female	0.315 (0.132)	0.018
MMP-8 (pg/ml)	7.563×10^{-6} (3.632×10^{-6})	0.039
MMP-9 (pg/ml)	-9.766×10^{-8} (2.234×10^{-7})	0.7

[1]For each regression model, square root transformation of the response variable (BASDAI or BAS-G) was carried out to achieve a normal distribution of the data.
