# Supplementary material for: Association of cytokine and matrix metalloproteinase profiles with disease activity and function in ankylosing spondylitis
Source: Arthritis Res Ther. 2012 May 28;14(3):R127. doi: 10.1186/ar3857 (PMC3446508; doi:10.1186/ar3857)
Supplement: Additional file 4 — Table S4 presenting levels of individual biomarkers comprising principal component 3 stratified by pack-year category. [file ar3857-S4.PDF]

Table S4. Levels of individual biomarkers comprising principal component 3 (PC3) stratified by pack year category

Variable (pg/ml)	Pack years 0 n = 61	1 - 15 n = 37	16 – 30 n = 17	>30 n = 14	p (trend)[1]
MMP-8	9750 (4767 – 14668)	12051 (7153 – 26796)	18674 (8263 – 24934)	26448 (14028 – 58829)	<0.0001
MMP-9	377000 (258488 – 607190)	527514 (332887 – 721161)	492148 (334153 – 820582)	447813 (280925 – 799118)	0.2
CXCL8	27.0 (21.1 – 40.5)	30.0 (21.3 – 51.8)	33.1 (21.9 – 72.5)	32.1 (20.1 -231.8)	0.2
HGF	421.7 (327.8 – 678.3)	437.6 (350.8 – 688.1)	390.9 (329.3 – 502.40	589.9 (354.1 – 984.4)	0.7

Median (interquartile range) values are shown. [1]Jonckheere-Terpstra test
