# Supplementary material for: Association of cytokine and matrix metalloproteinase profiles with disease activity and function in ankylosing spondylitis
Source: Arthritis Res Ther. 2012 May 28;14(3):R127. doi: 10.1186/ar3857 (PMC3446508; doi:10.1186/ar3857)
Supplement: Additional file 5 — Table S5 presenting a comparison of MMP levels in patient clusters with low or high MMP levels as selected by hierarchical cluster analysis. [file ar3857-S5.PDF]

Table S5. Comparison of MMP levels in patient clusters with low or high MMP levels as selected by hierarchical cluster analysis

Variable (pg/ml)	Low MMP cluster n = 94	High MMP cluster n = 63	p value[1]
MMP-1	2296 (1383 – 3345)	2778 (1545 – 4564)	0.026
MMP-2	146886 (126505 – 165298))	145022 (128628 – 168306)	0.6
MMP-3	14974 (11659 – 20537)	21452 (16611 – 30959)	<0.000001
MMP-8	8187 (4665 – 12546)	26357 (16731 – 33582)	<0.000001
MMP-9	302978 (229264 – 412972)	690892 (529103 – 836107)	<0.000001

Median (interquartile range) values are shown. [1]Mann-Whitney U test
